# Environmental Enrichment and Its Benefits for Migraine: Dendritic Cell Extracellular Vesicles as an Effective Mimetic

**Published:** 2021

**Authors:** Kae Myriam Pusic, Lisa Won, Richard Paul Kraig, Aya Darinka Pusic

**Affiliations:** Department of Neurology, The University of Chicago, Chicago, Il 60637-1470, USA

**Keywords:** Environmental enrichment, Enviromimetics, Migraine, Spreading depression, Extracellular vesicles, Exosomes, Oxidative stress, Interferon gamma

## Abstract

Environmental enrichment produces beneficial effects in the brain at genetic, molecular, cellular and behavior levels, and has long been studied as a therapeutic intervention for a wide variety of neurological disorders. However, the complexity of applying a robust environmental enrichment paradigm makes clinical use difficult. Accordingly, there has been increased interest in developing environmental enrichment mimetics, also known as enviromimetics. Here we review the benefits of environmental enrichment for migraine treatment, and discuss the potential of using extracellular vesicles derived from interferon gamma-stimulated dendritic cells as an effective mimetic.

## A Brief Overview of Migraine and Environmental Enrichment

Migraine is a common neurological disorder characterized by episodic intense and painful headaches that last between 4 and 72 hours. Typically, the headache is unilateral and pulsating, aggravated by physical activity, and often accompanied with nausea, dizziness, photophobia, hyperosmia, or phonophobia [1]. Migraine can be broadly grouped into two major subtypes, migraine without aura and migraine with aura. In migraine with aura, headaches are preceded by focal neurological symptoms that include transient visual, sensory, language or motor symptoms. Visual auras are by far the most common, and typically include scintillating scotomas – an arc of diminished vision edged by shimmering lights that travels slowly across the visual field [2]. Sensory auras are often experienced as numbness or tingling in one hand or on one side of the face. In its episodic form, migraines occur o to 14 days per month, whereas in patients with chronic migraine, headaches are experienced 15 or more days per month for three or more months [3].

Migraine and migraine modeled using spreading depression (SD) or systemic nitroglycerin injections are all associated with increased oxidative stress (OS). Agents that reduce OS show protective effects against migraine and migraine modeled in animals. The impact of OS in migraine models extends to nociceptive signaling within the trigeminal system, which is important to pain pathway activation in migraine [4-6]. OS in the trigeminal ganglion can increase expression of calcitonin gene-related peptide (CGRP) [7], a neuropeptide involved in development of migraine pain [8]. OS can also induce CGRP release from dorsal root ganglion neurons [6,9]. Newly developed anti-CGRP agents for migraine include small molecule CGRP receptor antagonists and CGRP monoclonal antibodies which inhibit or absorb circulating CGRP to block nociceptive activation of the trigeminal system [8]. Unfortunately, these existing therapeutic options to prevent migraine or mitigate the transformation of episodic to high-frequency and chronic migraine are only modestly effective [10]. However, environmental enrichment (EE) can reduce migraine frequency [11,12], decrease susceptibility to migraine modeled in rat [13], and alleviate chronic neuropathic pain concomitant with reduced spinal cord levels of CGRP [14].

EE consists of volitionally increased intellectual (i.e, learning and memory), physical and social activity, and has wide ranging physiological and behavioral effects, including enhancing cognition, memory, learning, behavior and motor coordination [15,16]. Preclinical studies have demonstrated the efficacy of EE as an intervention in an impressive number of neurological conditions, including but not limited to: Huntington’s disease [17], Parkinson’s disease [18], Alzheimer’s disease [19,20], traumatic brain injury [21], multiple sclerosis [22] and migraine [11,13]. EE has well-documented effects on immune function as well. In the first study of EE’s effects on immunity, Kingston and Hoffman-Goetz [23] postulated that EE exposure modulates immune reactivity, allowing for better regulation (a quicker response to stimuli, a more vigorous or efficient response, and a faster recovery to prestimulus levels). Much of this work has been conducted in the context of immunosenescence, as age is a critical factor in many neurological diseases (including chronic migraine). EE reverses the microglial dysfunction commonly seen in aging brains [24-27] and dampens pro-inflammatory responses from microglia and astrocytes [28].

While clinical studies of EE are more difficult to conduct, there is significant evidence that EE can produce beneficial effects in human patients as well. EE has been employed as a rehabilitation strategy for stroke survivors [29–30] and to reduce post-operative pain [31]. It is important to note that while prior studies have shown that the effects of voluntary exercise and those from exposure to an enriched environment are separable [32], they provide the greatest benefit when used simultaneously [33]. However, many clinical studies have focused on physical exercise or cognitive stimulation in isolation. Increased voluntary physical exercise has been linked to improved outcome for neurological diseases including depression [34], schizophrenia [35], epilepsy [36] and migraine [37]. Likewise, there have been numerous studies examining the role of intellectual enrichment in creating a ‘cognitive reserve’ that lessens the impact of brain disease on cognitive impairment [38,39]. Reports also show how social engagement and an active lifestyle can protect against dementia [40]. Notably, the Covid-19 pandemic has piqued increased interest in virtual forms of EE to improve cognitive health [41-43]. Taken together, these works provide evidence that EE has a beneficial impact on human health.

Though enrichment paradigms vary, they generally include the following aspects: 1) social enrichment, in which subjects are given increased exposure to conspecifics, 2) cognitive enrichment, which includes exposure to novel stimuli and experiential learning and 3) physical enrichment, consisting of voluntary exercise. An appropriate EE paradigm provides the opportunity to choose to engage in a variety of naturally rewarding activities in a non-stressful setting. Unfortunately, these criteria also present a major limitation. Not only does the complexity of applying a robust EE paradigm makes clinical use difficult, but practical implementation would require patients to have the physical and mental capacity to actively participate, which is not always possible. As a result, there has been increased attention to the development of EE mimetics or “enviromimetics” [44,45]. Conceivably, this approach would provide patients a means to exogenously access the benefits of EE until they are able to engage in an effective EE regimen on their own. Our lab has focused on studying the various mechanisms of EE-based mitigation of migraine (as modeled in rats) in an effort to identify potential mimetics.

## Spreading Depression as a Model of Migraine

SD is the most likely cause of migraine auras, and a likely cause of migraine pain through activation of the trigeminal pain pathway [4,46-48]. SD consists of increased synaptic activity followed by a period of electrical silence which slowly propagates through susceptible brain regions [47,49,50]. Following an episode of SD, neuronal excitability is temporarily elevated [51,52]. This increased excitability is accompanied by related increases in production of reactive oxygen species (ROS) [53,54].

SD also activates microglia and stimulates their release of cytokines, including tumor necrosis factor alpha (TNFα) and interferon gamma (IFNγ), suggesting a shift towards a M1-like pro-inflammatory state [55-59]. TNFα enhances synaptic efficacy by increasing membrane expression of excitatory α-amino-3-hydroxy-5-methyl-4-isoxazolepropionic acid receptors and decreasing membrane expression of inhibitory γ-aminobutyric acid-A receptors [60].

Another mechanism of increased susceptibility to recurrent SD is myelin damage/decompaction. Production of SD increases CNS production of pro-inflammatory cytokines (including IFNγ) and ROS, which converge upon activation of neutral sphingomyelinase 2 (nSMase2)-ceramide pathways. Myelin contains neutral sphingomyelinases [61] whose activity leads to sphingomyelin hydrolysis, ceramide formation and other downstream reactions with deleterious effects on myelin integrity. The net effect of this is a transient demyelination with SD that recovers over the course of a week [62]. Myelin disruption within grey matter may promote SD susceptibility by increasing hyperexcitability via ephaptic transmission (electrical crosstalk). This suggests a pathophysiological link behind the existing clinical evidence correlating disruption of myelin in patients suffering from migraine and multiple sclerosis, as multiple sclerosis also involves damage resulting from production of IFNγ by T cells, increased OS, glutathione depletion, and activation of nSMase2 [63-65].

Thus, SD creates a destructive feed-back cycle in which aberrant neuroexcitability, increased generation of ROS, production of pro-inflammatory cytokines and myelin damage caused by one episode of SD increases susceptibility to subsequent SD [53,54]. Frequent occurrences of SD without sufficient time for recovery may be responsible for the transition from episodic migraine to high-frequency and chronic migraine [66].

In contrast, physiological levels of ROS generated by intermittent increases in neuronal activity (e.g., from engaging in EE) leads to enhancement of antioxidant defenses [67]. This pattern of response is consistent with the hormetic model. The term “hormesis” describes a biphasic dose-response wherein exposure to a low dose of an irritant that would have negative consequences at a higher dose instead induces an adaptive response that is beneficial [68]. It is a well-conserved response pattern that has been observed at the cellular, tissue and organismal level [69], and appears to apply to mechanisms of EE-mediated neuroprotection.

## Mechanisms Involved in EE-based Mitigation of SD

Insulin-like growth factor-1 (IGF-1) is a hormone that can modulate neuronal plasticity, survival and proliferation [70]. It is a major mediator of the neuroprotective effects of exercise [71], and its expression levels increase with EE [72]. While most circulating IGF-1 is produced by the liver, EE has been shown to increase brain uptake of IGF-1 from the periphery [73]. IGF-1 is protective against SD *in vitro* in hippocampal slice cultures [53,54]. This effect involves decreasing post-SD levels of TNFα and decreased production of ROS from microglia; two factors that can contribute to the neuronal hyperexcitability that promotes recurrent SD [53,54]. Furthermore, nasally administered IGF-1 reduces SD susceptibility and OS-induced trigeminal nociceptive activation (i.e., reduces CGRP expression) in two animal models of migraine, SD and systemic injection of nitroglycerin [4,5,74].

Interleukin-11 (IL-11) is an anti-inflammatory cytokine with immunomodulatory and neuroprotective properties [75-78]. Rats exposed to EE have increased levels of neuronal IL-11, perhaps reflective of phasically increased neuronal activity from learning [15]. IL-11 significantly reduces TNFα expression, including that produced following SD. When nasally administered to rats, IL-11 reduced susceptibility to SD, significantly reduced protein carbonylation (reflective of oxidative damage) and promoted an M2a-skewed microglial phenotype [13]. The M2a phenotype is associated with suppression of inflammation, and has increased surface expression of Arginase-1. This is of note here, as Arg-1 mediates the conversion of arginine to polyamines, proline and orthinines, and outcompetes inducible nitric oxide synthase (iNOS) for access to arginine (also the substrate for nitric oxide production [79]) to reduce production of ROS.

IFNγ is a highly pleotropic cytokine with diverse functions in both innate and adaptive immunity and host defense. While commonly considered a pro-inflammatory cytokine, it is also involved in regulation of anti-inflammatory responses, and may be better described as an immunoregulatory effector molecule [80]. IFNγ activates intracellular molecular signaling networks that modulate the transcription of hundreds of genes and mediate numerous biological responses [81]. It is likely involved in the low level inflammatory signaling of EE that acts as a mild stressor to promote adaptive responses [82]. In support of this, studies have shown that regular moderate exercise increases IFNγ levels in human plasma [83], and that IFNγ is involved in spatial learning [84]. Though its role in multiple sclerosis and other degenerative disorders is contested and largely thought to be detrimental, studies have shown that this is disease stage and context dependent [85]. Pre-exposure of healthy brain to IFNγ reduces subsequent demyelination in animal models of multiple sclerosis [86-90]. Likewise, IFNγ plays dual roles in modulation of SD susceptibility. Though elevated IFNγ and associated increases in OS are detrimental in SD, in accordance with physiological conditioning hormesis, phasic exposure to IFNγ instead produces an adaptive response that is protective against SD and prevents the associated myelin damage. Treatment with a single 12 hour pulse of IFNγ (or phasic IFNγ treatment for a week) emulates the phasic changes of EE and likewise increases myelination, reduces susceptibility to SD and reduces OS [91]. This effect is likely mediated by release of extracellular vesicles from IFNγ-stimulated microglia, and is in line with work showing that activation of microglia with low concentrations of IFNγ induces a protective anti-inflammatory (M2a-like) phenotype [92].

## IFNγ Stimulated Dendritic Cell Extracellular Vesicles as an EE-mimetic

Extracellular vesicles (EVs) are lipid membrane vesicles that are secreted by many cell types and are involved in a multitude of functions, both physiological and pathological [93]. EVs have the potential for targeting specific cell types to deliver their cargo of protein, mRNA and miRNA. This cargo is protected from degradation by proteases and RNases while the vesicle is in the interstitial space, and retains bioactivity once taken up by a recipient cell. In this way, EVs facilitate interactive signaling between cells. EVs are non-toxic and do not provoke adverse immune reactions, and can be easily nasally delivered to the brain - traits that support the potential utility of EV-based therapies. The specific composition of their cargo is influenced by their parent cell type and disease and/or activation state. EVs can be isolated from biofluids or conditioned medium by one of a variety of methods that yield an EV enriched preparation that likely represents a heterogeneous mixture of exosomes (30-150 nm diameter), microvesicles (200-1000 nm diameter), apoptotic bodies and protein complexes [94]. EVs isolated from the serum of rats exposed to EE significantly increase myelin content and oligodendrocyte precursor cell levels, and reduce OS in hippocampal slice cultures and when nasally administered to naive rats [95]. Importantly, exposure of an aged animal to EE restores their ability to produce myelination-promoting EVs, suggesting that EE-induced reversal of age-related immune dysfunction may be involved.

To mimic EE, we stimulated primary dendritic cell cultures with low-level IFNγ. EVs produced in this way (IFNγ-DC-EVs) increase myelination and oxidative tolerance *in vitro* and *in vivo* [96]. Treatment with IFNγ-DC-EVs significantly reduces SD susceptibility *in vitro* and *in vivo*, reduces microglial M1 product iNOS, and reduces OS-mediated damage [97]. IFNγ-DC-EVs appear to impact all pathways identified above that contribute to EE-mediated modulation of SD susceptibility. 1) IFNγ-DC-EVs reduce OS and increase glutathione. This is perhaps due to their containing miRNA species involved in resolution of inflammation and reduction of OS. For example, IFNγ-DC-EVs contain miR-532-5p which reduces microglial expression of pro-inflammatory cytokines in response to lipopolysaccharide [98] and suppresses NADPH oxidase 2 expression to reduce ROS production [99]. They also contain miR-181a, which dampens pro-inflammatory signaling and reduces production of ROS in macrophages/monocytes [100] and similarly regulates inflammation in the CNS [101]. 2) IFNγ-DC-EVs reduce microglial markers of M1 polarization. Another enriched miRNA, miR-124, increases anti-inflammatory signaling and downregulates M1-associated IL-6, TNFα and iNOS [102,103]. This is in line with our finding that microglial polarization state impacts SD with M1-like pro-inflammatory microglia increasing susceptibility. It is likely that IFNγ-DC-EVs produce a similar effect to EE-induced changes in microglial polarization via neuronal-activity-induced increased production of IL-11. 3) IFNγ-DC-EVs promote myelination. Like phasic treatment with low-level IFNγ, IFNγ-DC-EVs promote myelin production, likely through delivery of miR-219 which is necessary for myelination. Taken together, this suggests IFNγ-DC-EVs may be a potent EE-mimetic for use in migraine treatment. It is important to note that EVs are non-toxic and can easily cross the blood brain barrier without use of an additive vehicle.

Although most studies of the benefits of EE on the central nervous system have been conducted in rodents, there is ample evidence that EE is effective in a wide variety of other species including but not limited to fish [104,105], birds [106,107], rabbits [108,109], ferrets [110], pigs [111], dogs [112], octopi [113], and marmosets [114]. As detailed above, there is evidence that EE is likely beneficial for CNS health in humans as well. Additionally, miRNAs show high levels of conservation even between distantly related species [115]. Thus, it is likely that IFNγ-DC-Exos will function as an EE-mimetic in humans as well. However, while the biological function of EVs can be well-conserved between species [116], xenogenic EVs may initiate the synthesis of foreign proteins in recipient cells [117] and potentially trigger adverse immune reactions. Accordingly, proof of efficacy of human EVs (hIFNγ-DC-EVs) is important to development of IFNγ-DC-EVs for use as an enviromimetic.

## Exploring the Potential of Human-derived IFNγ-DC-EVs

Fresh human bone marrow aspirates that have been verified to be pathogen-free were obtained from Lonza (Walkersville, MD) for differentiation into dendritic cells. Monocytes were isolated via density centrifugation with Lymphocyte Separation Medium (Lonza), and CD34^+^ cells were purified by magnetic cell isolation (Miltenyi Biotec). Cells were maintained in RPMI+10% fetal bovine serum (FBS) supplemented with a standard complement of cytokines for 10 days of culture. At day 10, cells were transferred to RPMI+10% exosome-depleted FBS (System Biosciences) containing the same cytokines, with or without IFNγ. At day 13, conditioned medium was harvested from unstimulated or IFNγ-treated dendritic cells for EV isolation.

**Figure 1:**
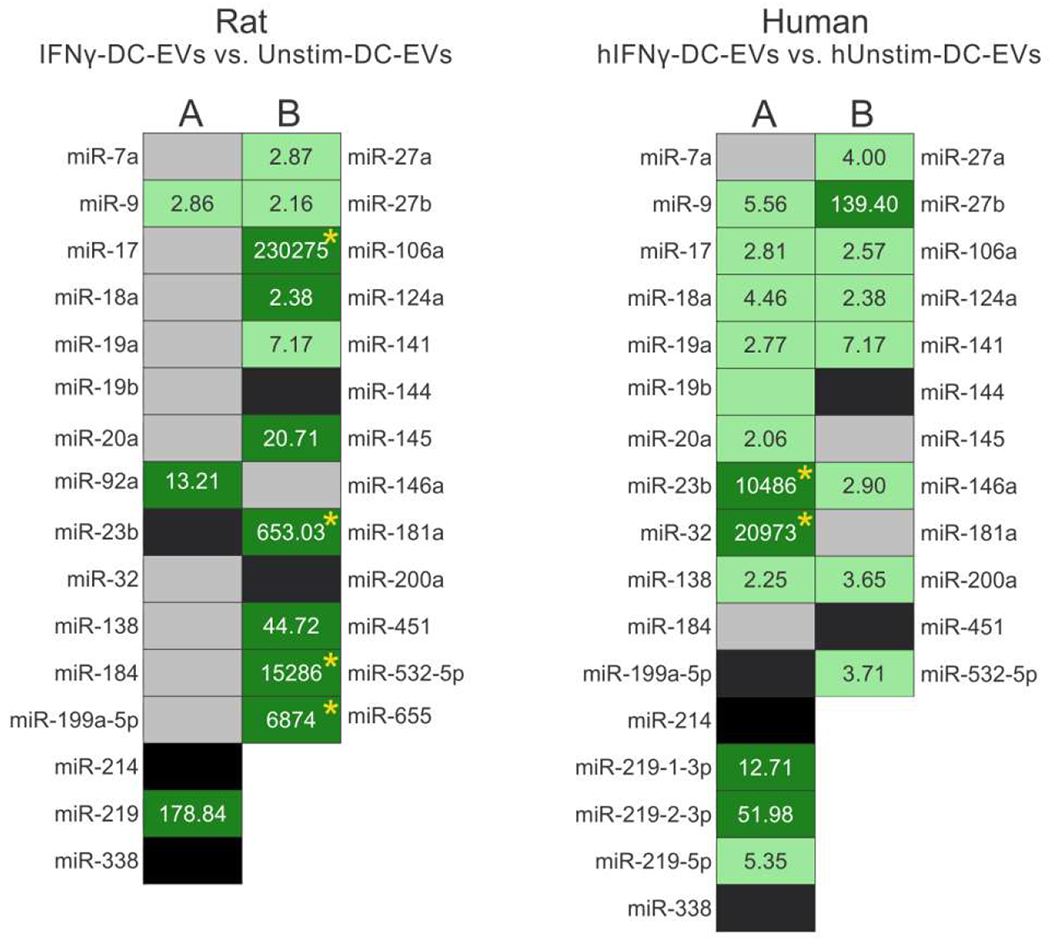
miRNA expression profile of rat and human IFNγ-DC-EVs. Total RNA was extracted from EVs using the mirVana miRNA Isolation Kit (Life Technologies). cDNA was synthesized from total RNA using Megaplex RT primers and TaqMan miRNA RT kits, followed by preamplification with Megaplex PreAmp Primers (all Life Technologies). Samples were loaded to TaqMan microfluidic cards (Rodent Micro RNA A+B Cards Set V3.0 or Human MicroRNA A+B Cards Set V3.0 as appropriate; Life Technologies) and run on an Applied BioSystems 7900HT thermocycler (University of Chicago Genomics Core Facility). All procedures above were performed according to manufacturer’s instructions. All analyses included two technical replicates per biological sample, and were performed via the comparative Ct method. Endogenous controls included U6 snRNA, RNU43 snoRNA, and U1 snRNA. Greater than 2-fold change was considered significant. In some cases (denoted with an asterix *) fold change calculations could not be accurately made due to the miRNA species not being present in the unstimulated condition. In these situations, Ct values were set at 35 (our cycling protocol consists of a total of 40 amplification cycles). Results show expression levels of specific miRNAs involved in **(A)** myelin production/oligodendrocyte differentiation, and **(B)** anti-inflammatory responses. (Left) miRNA content of rat IFNγ-DC-EVs were compared to that of EVs from unstimulated rat dendritic cell EVs (Unstim-DC-EVs). (Right) miRNA content of interferon gamma-stimulated human EVs (hIFNγ-DC-EVs) were compared to that of EVs from unstimulated human dendritic cell EVs (hUnstim-DC-EVs). Black panels indicate mature miRNA species that could not be detected; grey panels indicate miRNAs that were readily detectible but not significantly enriched; light green indicate significantly enriched (i.e., >2 fold) miRNAs; and dark green indicates very highly enriched (i.e., >10 fold) miRNAs. Rat data was adapted from a previous publication [13].

As a first measure of the feasibility of producing human EE-mimetic EVs, their miRNA contents were profiled. Here we show that hIFNγ-DC-EVs contain a similar cohort of miRNA species as rat IFNγ-DC-EVs (Figure 1). Importantly, miRNA species found to play functional roles in rat IFNγ-DC-EVs were similarly upregulated in hIFNγ-DC-EVs. While future studies to explore their functional effects are necessary, this begins to suggest that human-derived dendritic cells respond to IFNγ in a similar way as rat dendritic cells and may indeed produce similarly beneficial EVs.

In conclusion, there is a need to harness the beneficial effects of EE for treatment of migraine and we believe hIFNγ-DC-EVs have great potential for development as an effective mimetic that can be easily nasally administered to the brain.
